# PET imaging utilization and trends in Germany: a comprehensive survey

**DOI:** 10.1007/s00259-025-07323-x

**Published:** 2025-05-03

**Authors:** Adrien Holzgreve, Dirk Hellwig, Henryk Barthel, Ambros J. Beer, Carsten Kobe, Constantin Lapa, Matthias Miederer, Sarah Schwarzenböck, Robert Seifert, Andrei Todica, Ken Herrmann, Frank M. Bengel, Michael Schäfers, Detlef Moka, Markus Luster, Wolfgang P. Fendler

**Affiliations:** 1 PET Committee of the German Society of Nuclear Medicine, Göttingen, Germany; 2 Department of Nuclear Medicine, LMU University Hospital, LMU Munich, Munich, Germany; 3 Ahmanson Translational Theranostics Division, Department of Molecular and Medical Pharmacology, David Geffen School of Medicine, University of California Los Angeles (UCLA), Los Angeles, CA USA; 4 Department of Nuclear Medicine, University Hospital Regensburg, Regensburg, Germany; 5 Department of Nuclear Medicine, Leipzig University Medical Centre, Leipzig, Germany; 6 Department of Nuclear Medicine, University Hospital Ulm, Ulm, Germany; 7 Department of Nuclear Medicine, University Hospital Cologne, Cologne, Germany; 8 Nuclear Medicine, Faculty of Medicine, University of Augsburg, Augsburg, Germany; 9 Department of Translational Imaging in Oncology, National Center for Tumor Diseases (NCT/UCC) Dresden, Faculty of Medicine and University Hospital Carl Gustav Carus, University of Technology Dresden (TUD), Helmholtz-Zentrum Dresden-Rossendorf (HZDR), Dresden, Germany; 10 German Cancer Research Center (DKFZ), Heidelberg, Germany; 11 Department of Nuclear Medicine, University Medical Center Rostock, Rostock, Germany; 12 Department of Nuclear Medicine, University Hospital Bern, Bern, Switzerland; 13 DIE RADIOLOGIE, Munich, Germany; 14 Department of Nuclear Medicine, University Hospital Essen, Essen, Germany; 15 Department of Nuclear Medicine, Hannover Medical School, Hannover, Germany; 16 Department of Nuclear Medicine, University Hospital Münster, Münster, Germany; 17 Nuclear Medicine Centre, Essen, Germany; 18 Department of Nuclear Medicine, Philipps University of Marburg, Marburg, Germany

**Keywords:** Positron emission tomography (PET) volume, Germany, European union (EU), Survey, Clinical practice and health policy

## Abstract

**Introduction:**

PET imaging is a key diagnostic procedure in clinical routine worldwide. While public figures on PET volume are available in many countries, until now these numbers were not publicly known for Germany.

**Methods:**

On behalf of the PET committee of the German Society of Nuclear Medicine, we conducted a comprehensive survey among PET centers in Germany to collect data on PET imaging, including the total PET volume and indication groups.

**Results:**

National total PET volume in 2021 was 154,400 scans (94% PET/CT, 6% PET/MRI). PET volume in 2021 normalized to total population was lower in Germany (1,857 scans per 1 million inhabitants) when compared to public figures from France (10,182 scans), Belgium (9,866 scans), or Italy (4,312 scans). PET volume in Germany demonstrated significant growth 2017 to 2021 (+ 48%). Top three indication fields were oncological (re)staging (76%), theranostic (13%), and neurology (4%). The top three indications were lung cancer (31%), prostate cancer (16%), and lymphoma/leukemia (12%). The top three radiotracers used were [^18^F]FDG (75%), PSMA radioligands (17%), and somatostatin-receptor radioligands (8%).

**Conclusions:**

Clinical adoption of PET imaging in Germany is behind compared to Italy, France, and Belgium. However, newly established outpatient reimbursement seems to contribute to recent growth in PET volume. We observe considerable shift towards theranostic applications.

**Supplementary Information:**

The online version contains supplementary material available at 10.1007/s00259-025-07323-x.

## Introduction

PET imaging is a key diagnostic procedure in clinical routine, particularly in the field of oncology, and it is gaining ground worldwide [1, 2]. While public figures on PET volume are available in many countries, until now these numbers have been lacking for Germany, which is a driver of theranostic research and early clinical adopter. PET reimbursement in Germany has changed drastically since the early 2000 years. After a temporary total loss of reimbursement for PET in statutory health care and subsequently slow recognition of individual indications, lengthy negotiations for implementation of a nationwide reimbursement for PET in Germany failed, and eventually multilayer alternative reimbursement routes emerged. These include outpatient routes such as specialist medical care (ASV) oncology programs, private insurance and the self-pay sector, direct agreements between health insurance companies and providers, and others. Thus, we now aimed at collecting data on current and past clinical PET operation in a comprehensive survey conducted among PET centers in Germany.

## Materials and methods

**Fig. 1:**
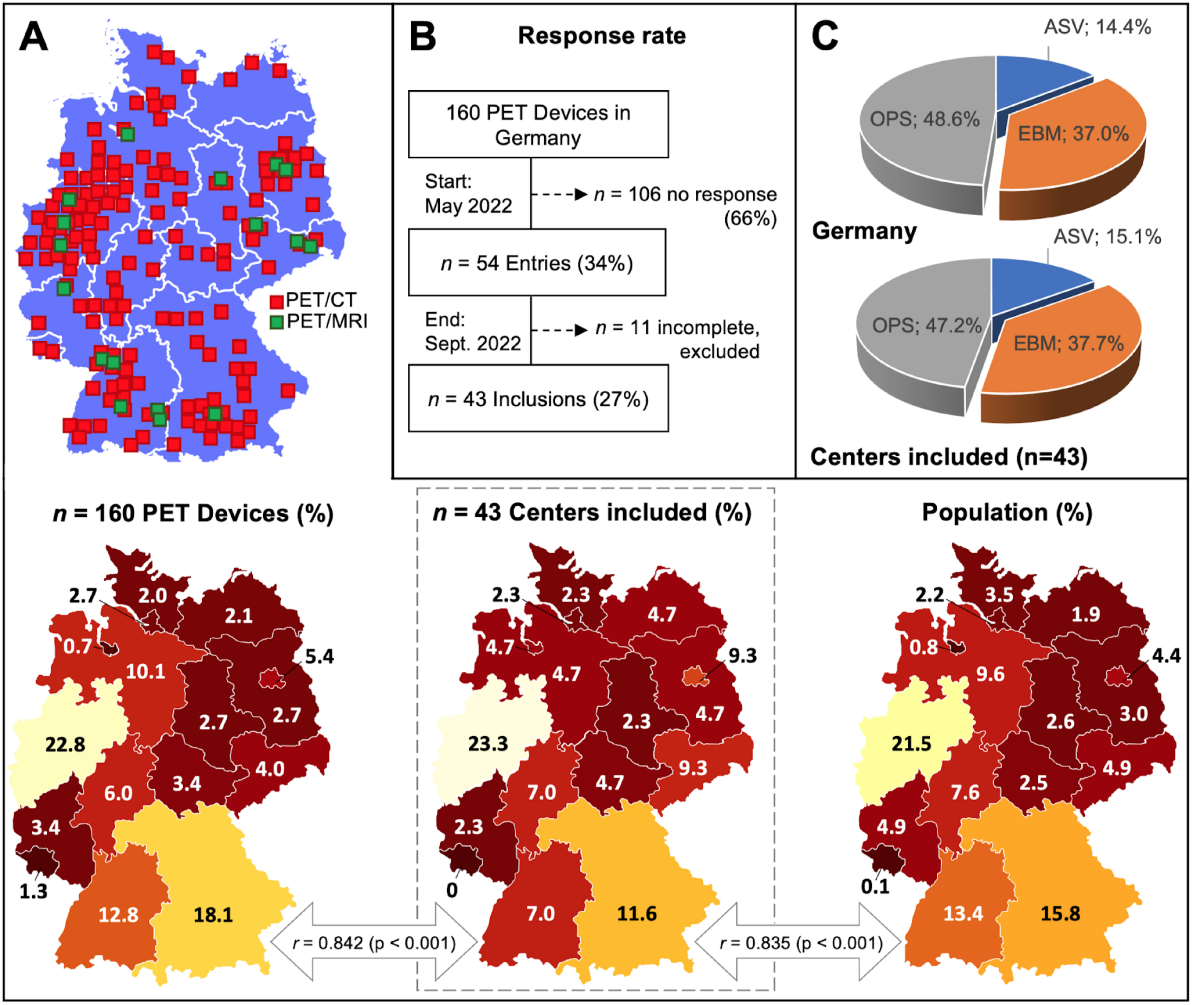
Distribution of German sites and population. (**A**) Regional distribution of PET centers included in the analysis in correlation to all PET centers in Germany and to the German population, Pearson’s correlation was used to analyze their linear relationship; (**B**) Response rate; (**C**) Proportion of reimbursement types in the survey vs. national statistics. Correlation of proportions on the level of the sixteen states in Germany shows that the distribution of PET centers included in the analysis is highly representative of the distribution of all PET centers in Germany (*r* = 0.842, *p* < 0.001), and of the population distribution in Germany (*r* = 0.835, *p* < 0.001). ASV indicates *Ambulante Spezialfachärztliche Versorgung (outpatient specialized care)*; EBM, *Einheitlicher Bewertungsmaßstab (outpatient care)*; OPS, *Operationen- und Prozedurenschlüssel (inpatient care)*

A survey was developed by the PET committee of the German Society of Nuclear Medicine (DGN) using SurveyMonkey (San Mateo, CA, US). Questions were distributed online by the offices of the German Nuclear Medicine Associations *Deutsche Gesellschaft für Nuklearmedizin* (DGN) and *Berufsverband Deutscher Nuklearmediziner* (BDN) among 160 PET centers. Overview of PET centers was obtained from the German Electro and Digital Industry Association (ZVEI e. V.), see Fig. 1A. Data collection took place 05/01/2022–09/30/2022 and covered PET scans performed 01/01/2021–12/31/2021 in hospitals, the ambulatory setting, and research institutions. Overall, 43 centers (see Table 1) operating a total of 60 PET devices (see Table 2) responded with complete survey entries (27% response rate). Survey response is presented in Fig. 1B. Main items of the survey included PET volume, indication groups, specific oncological entities, PET radioligands, reimbursement paths, among others, while covering both clinical and research activity. The survey is attached in the Supplement. Detailed results on national reimbursement routes are beyond the scope of this publication and will be made available to the German nuclear medicine community separately.

**Table 1:** PET centers included (*n* = 43)

Type of Center (*n* = 43)	*n* (%)
Academic Research Hospital	21 (49%)
Public Hospital	8 (19%)
Private Hospital	3 (7%)
Outpatient Healthcare Center	3 (7%)
Private Practice	7 (16%)
Research Institution	1 (2%)
Number of PET (PET/CT or PET/MRI) Devices per Center	
4 Devices	1 (2%)
3 Devices	3 (7%)
2 Devices	8 (19%)
1 Devices	31 (72%)

**Table 2:** Devices included (*n* = 60)

Type of Device	*n* (%)
PET/CT	50 (83%)
PET/MRI	10 (17%)
EARL Accreditation	
Yes	18 (30%)
No	36 (60%)
Unknown	6 (10%)

EARL = European Association of Nuclear Medicine (EANM) Research GmbH Accreditation Program

To calculate overall PET volume in Germany, survey results were complemented by the frequency statistics of the National Association of Statutory Health Insurance Funds (“GKV-Frequenzstatistik”; outpatient specialized care “ASV” reports) and the national hospital statistics of the German Federal Statistics Office (“Krankenhausstatistik”, DESTATIS) each for 2021. Using these additional sources, survey results were scaled to the national level. Figure 1 (A, C) illustrates that survey results were representative of the nationwide distribution for PET devices, centers and population as well as reimbursement paths. The German figures were compared with those of neighboring European Union countries whose professional nuclear medicine societies provided comparative national data.

## Results

**Fig. 2:**
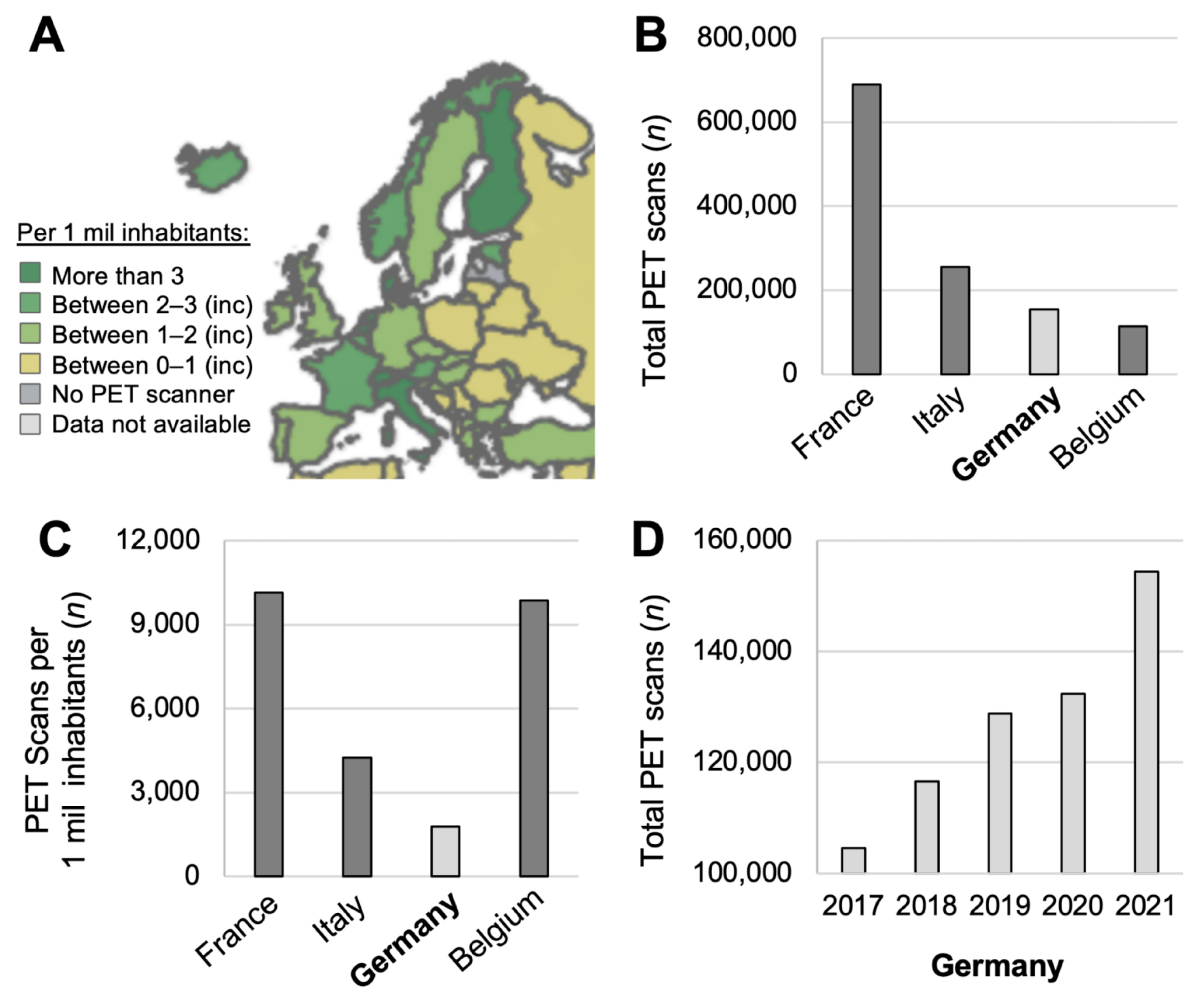
PET devices and volume in Germany and selected European Union nations. (**A**) PET device ranges per country, adopted with permission form the International Atomic Energy Agency Database, IMAGINE; Germany reports between 1 and 2 PET devices per 1 million inhabitants [24]; (**B**) Total number of PET scans per year [25, 26]; (**C**) Number of PET scans per 1 million inhabitants per year [25, 26]; (**D**) Number of PET scans 2017–2021 in Germany

In total, 154,400 PET scans were performed in 2021 in Germany. PET volume in Germany is low compared to France, Belgium or Italy. The PET devices and volumes per nation are displayed in Fig. 2A-C. 17% of devices were PET/MRI that contributed 6% of the overall PET volume. PET volume in Germany demonstrates significant growth over 5 years (+ 48%), see Fig. 2D.

**Fig. 3:**
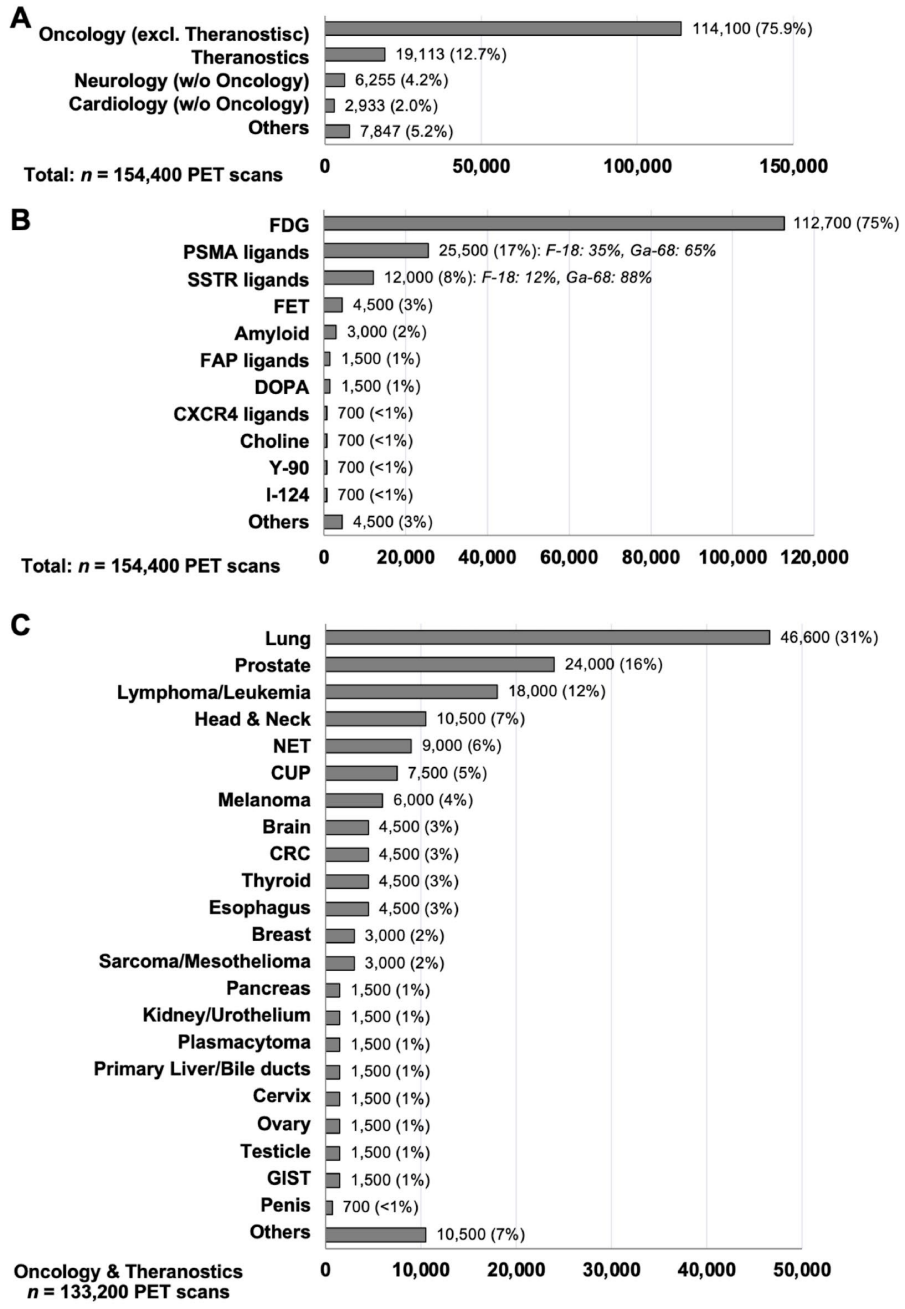
Overview of PET indications in Germany in 2021. (**A**) Indication groups. The top three other indication groups were fever and inflammation of unknown origin, rheumatic disorders, parathyroid imaging. (**B**) Radioligands. Top three other radioligands were C-11 methionine, O-15 water, and tau radioligands. (**C**) Oncological entities. The top three other oncological entities were dermatologic, gynecologic, and thoracic malignancies. NET indicates *Neuroendocrine Tumor*; CUP, *Cancer of Unknown Primary*; CRC, *Colorectal Cancer*; GIST, *Gastrointestinal Stromal Tumor*

Oncologic (re-)staging and patient selection in a theranostic setting accounted for 88.6% of all PET scans. The top five oncological indications were lung cancer (31%), prostate cancer (16%), lymphoma/leukemia (12%), head and neck cancers (7%), and neuroendocrine tumors (6%). An overview of all PET indications in Germany including distinct oncology and theranostic indications is given in Fig. 3. The top five PET radiotracer groups were 2-[^18^F]fluoro-2-deoxy-D-glucose ([^18^F]FDG) (75%), prostate-specific membrane antigen (PSMA) radioligands (17%), somatostatin-receptor (SSTR) radioligands (8%), amino acid analogs (3%), and amyloid radioligands (2%). A list of compounds for whom centers held a manufacturing license with permission for use as an investigational medicinal product in clinical trials is given in Supplementary Table 1. Five centers each had a license for ^68^Ga-PSMA ligands and for ^68^Ga-SSTR ligands, and two centers each had a license for ^18^F-PSMA ligands, FAP radioligands, amyloid radioligands, [^18^F]FET, and ^15^O water.

## Discussion

We present data on PET use in a comprehensive survey conducted among imaging centers in Germany and for the first time calculated and compared the total PET volume in Germany. Compared to other European industry nations, clinical adoption of PET in Germany was lower in term of total volume, relative volume and number of PET devices.

The complex reimbursement history is likely a key contributor to the German backlog in PET availability. In 2002, a total loss of reimbursement for PET in statutory health care was decided by federal institutions [3, 4]. In the same year, the Deutsche Forschungsgemeinschaft (DFG, German Research Foundation) as the major funding institution decided to approve the installation of no more than five combined PET/CT devices at German university hospitals [5]. In December 2004, a discussion of several university hospitals in Germany with the DFG eventually led to the general eligibility of PET/CT installation to be supported by public funds. Meanwhile, substantial investments in PET/CT purchases were made in other countries (e.g., resulting in 1.35 vs. 0.18 PET/CT devices per 1 million inhabitants in the USA vs. Germany in 2005 [5]). Temporary exclusion of PET device reimbursement from DFG’s large-scale equipment initiatives contributed to aggravate this situation, despite one of the major PET-vendors being located in Germany, and despite having the highest health care expenditure relative to gross domestic product within Europe. In December 2005, PET reimbursement in statutory health care was eventually recognized by federal institutions, however only for three oncologic indications: Staging of primary non-small cell lung carcinoma, detection of recurrence (in case of reasonable suspicion) in primary non-small cell lung carcinoma, and characterization of indeterminate lung nodules [6, 7]. German health care providers subsequently applied for an extension of reimbursement to cover various PET indications. Further outpatient indications were rejected by the Federal Joint Committee (“*Gemeinsamer Bundesausschuss*”, G-BA) [8]. Ultimately, all ongoing applications for PET reimbursement were withdrawn in 2018 except for lung cancer, head and neck tumors, and lymphoma. An interim agreement to evaluate the benefits of PET/CT in recurrent colorectal carcinoma also failed. After a lengthy deadlock in negotiation and despite strong implementation of PET in national and international clinical guidelines, on November 20, 2020 G-BA eventually announced that the use of PET has not proven sufficiently beneficial for patients, which would have been a prerequisite for nationwide reimbursement [9]. Meanwhile, PET/CT was increasingly included in outpatient specialist medical care (ASV) oncology programs since 2014 [10]. Facilitated by new reimbursement possibilities [4], PET volume in Germany increased significantly 2017 to 2021. This is in line with PET growth in other member countries of the Organisation for Economic Co-operation and Development (OECD) [2]. However, PET-reimbursement outside ASV is limited in Germany and direct agreements between health insurance companies and providers were opened for many regions.

The data collected in this survey imply further growth potential of PET in Germany: As of 2021, “theranostics” has already been the second most important indication group, and it can be expected that this group in particular will continue to grow due to the approval of PSMA radiopharmaceutical therapy by both the U.S. Food and Drug Administration and European Medicines Agency in 2022 [11]. These developments are also reflected by the tracers used: PSMA radioligands and SSTR radioligands were the second and third most common tracers, respectively. In addition, new promising theranostic agents are on the rise and will further expand this sector. These are based on novel theranostic targets such as Fibroblast activation protein and carbonic anhydrase IX [12–14, 15] as well as on the use of alternate radionuclides including the alpha emitters actinium-225 and lead-212 or the short-range conversion and Auger electron emitter terbium-161 [16–18]. Academic sites in Germany played an important role in the clinical translation of novel theranostic applications including initial development of PSMA radioligand therapy [19, 20]. Data collected in our survey reveal that multi-center PET imaging trials are feasible in Germany, as several centers hold licenses for investigational use in clinical trials for [^18^F]FDG, PSMA radioligands, SSTR radioligands, and other compounds.

Of note, PET/MRI accounted for a small proportion of performed scans (6%), despite significant availability of devices (17% of all PET devices in the survey). However, it must be acknowledged that PET/MRI devices are not necessarily expected to achieve high throughput rates and that their availability in Germany has been driven by dedicated research funding outside of clinical routine.

Focusing on the German system alone falls short of explaining international differences in PET availability and utilization. Both geomedical and regulatory particularities of national health care systems may contribute to diverging PET figures per capita. For instance, the criteria by which national decision-making authorities approve a new diagnostic procedure vary across countries. Whereas in the USA an observed impact of PET on the therapeutic decision-making may facilitate approval, German authorities often required results of prospective trials demonstrating an impact of PET on patient-related outcomes such as improved overall survival for implementation of PET in official guidelines, contributing to overall longer timescales from the introduction of an innovative imaging procedure to marketing authorization. Different stakeholders including representatives of hospitals and of physicians take part in the negotiations and decision process of G-BA in Germany and may therefore directly exert influence on the approval process whereas in other highly regulated health care systems the direct influence of lobby groups tends to be more restricted. The impact of these interrelationships on the total PET volume cannot be measured, but it is reasonable to assume that they have played a key role in shaping resource allocation in Germany. Beyond regulatory aspects, regional cancer statistics put the survey results further into perspective. Consistently in all four countries Germany, France, Belgium, and Italy, prostate cancer as a main PET indication was the top-ranked cancer by absolute number of incident cases for all ages in males; tracheal, bronchus, and lung cancer as another main PET indication was the top-ranked cancer by absolute number of deaths for all ages in males; and breast cancer was the top-ranked cancer by absolute number of incident cases and deaths for all ages in females [21]. Non-Hodgkin lymphoma as another main PET indication was ranked 11^th^ regarding mortality across all cancers in each Germany, France, Belgium, and Italy [21]. On a global note, the number of PET scans per 1 million inhabitants was lower in Germany (1,857) compared to 2019/2020 data of Australia (4,600), the United Kingdom (3,500), and Canada (3,300) [22]. Reasons for the decision to publish the study results in a European rather than in a German journal include that the results are especially informative in this international context given that Germany is considered a leader in Nuclear Medicine.

Here we have surveyed clinical PET operation in Germany. Findings allow conclusions for reimbursement policy and may thereby influence the health policy in Germany. On the one side, our data suggest that restrictive reimbursement and the framework of device funding may impede PET availability. Yet, further potential factors with an influence on PET usage statistics have to be considered, such as regional cancer statistics or the educational levels of referring physicians. On the other side, theranostic applications demand PET and their fast growth will likely further increase the demand of PET examinations.

Limitations include a response rate that appears to be sub-average for online surveys. Yet, a 20–25% response rate is assumed to provide fairly confident estimates in surveys with a smaller sample size (i.e., *n* < 500, as in this survey) [23], which is in line with our response rates (34% including incomplete entries, 27% complete data entries). The lack of data completeness also raises questions about the representativity of the available data set. Yet, it must be pointed out that survey results were representative of the nationwide distribution for PET devices, centers, and population, as well as of relative reimbursement paths. A high proportion of PET devices in Germany is installed at university hospitals and consequently, a high proportion of the responses came from academic institutions. Yet, half of responses coming from academic institutions in the survey may over-represent this sector compared to the national distribution with potential influence on estimates in the analysis, e.g. for indication groups such as in the theranostic field. The use of data derived from both public health statistics and survey responses needed to be combined to account for unrecorded PET scans in the areas of research, the private insurance and the self-pay sector. This approach enabled the most comprehensive data available to be presented so far to give an overall impression of the current PET landscape in Germany.

In conclusion, in a comparison with three neighboring countries, PET adoption in Germany is behind, possibly for multifactorial reasons including previous and ongoing restrictive reimbursement policy. New outpatient reimbursement as well as theranostic indications may have triggered recent growth in PET volume. PET/CT for oncology is the main driver of PET operation in Germany with considerable focus on theranostic applications.

## Electronic supplementary material

Below is the link to the electronic supplementary material.

Supplementary Material 1

## Data Availability

The datasets generated during and/or analyzed during the current study are not publicly available due to guaranteed privacy to survey participants but some aggregated or unlabeled/anonymized data may be available from the corresponding author on reasonable request.
