# Supplementary material for: PET imaging utilization and trends in Germany: a comprehensive survey
Source: Eur J Nucl Med Mol Imaging. 2025 May 3;52(12):4390–8. doi: 10.1007/s00259-025-07323-x (PMC12491347; doi:10.1007/s00259-025-07323-x)
Supplement: Supplementary file 1 — Supplementary Material 1 [file 259_2025_7323_MOESM1_ESM.docx]

**PET Imaging Utilization and Trends in Germany: A Comprehensive Survey**

Adrien Holzgreve ^1,2,3^, Dirk Hellwig ^1,4^, Henryk Barthel ^1,5^, Ambros J. Beer ^1,6^, Carsten Kobe ^1,7^, Constantin Lapa ^1,8^, Matthias Miederer ^1,9^, Sarah Schwarzenböck ^1,10^, Robert Seifert ^1,11^, Andrei Todica ^1,2,12^, Ken Herrmann ^13^, Frank M. Bengel ^14^, Michael Schäfers ^15^, Detlef Moka ^16^, Markus Luster ^17^, Wolfgang P. Fendler ^1,13^

1. PET Committee of the German Society of Nuclear Medicine
2. Department of Nuclear Medicine, University Hospital, LMU Munich, Munich, Germany
3. Ahmanson Translational Theranostics Division, Department of Molecular and Medical Pharmacology, David Geffen School of Medicine, University of California Los Angeles (UCLA), Los Angeles, CA, USA
4. Department of Nuclear Medicine, University Hospital Regensburg, Regensburg, Germany
5. Department of Nuclear Medicine, Leipzig University Medical Centre, Leipzig, Germany
6. Department of Nuclear Medicine, University Hospital Ulm, Ulm, Germany
7. Department of Nuclear Medicine, University Hospital Cologne, Cologne, Germany
8. Nuclear Medicine, Faculty of Medicine, University of Augsburg, Augsburg, Germany
9. Department of Translational Imaging in Oncology, National Center for Tumor Diseases (NCT/UCC) Dresden: Faculty of Medicine and University Hospital Carl Gustav Carus, University of Technology Dresden (TUD), German Cancer Research Center (DKFZ) Heidelberg, and Helmholtz-Zentrum Dresden-Rossendorf (HZDR), Germany
10. Department of Nuclear Medicine, University Medical Center Rostock, Rostock, Germany
11. Department of Nuclear Medicine, University Hospital Bern, Bern, Switzerland
12. DIE RADIOLOGIE, Munich, Germany
13. Department of Nuclear Medicine, University Hospital Essen, Essen, Germany
14. Department of Nuclear Medicine, Hannover Medical School, Hannover, Germany
15. Department of Nuclear Medicine, University Hospital Münster, Münster, Germany
16. Nuclear Medicine Centre, Essen, Germany
17. Department of Nuclear Medicine, Philipps University of Marburg, Marburg, Germany

First and Corresponding Author:

Adrien Holzgreve, MD, MHBA

Ahmanson Translational Theranostics Division, Dept. of Molecular and Medical Pharmacology, David Geffen School of Medicine, University of California Los Angeles (UCLA)

Peter Morton Medical Building, 200 Medical Plaza, Suite B-114, Los Angeles, CA 90095-7370

Phone: +1 310-794-1005, Fax: +310-267-0227, E-Mail: [AHolzgreve@mednet.ucla.edu](mailto:AHolzgreve@mednet.ucla.edu)

**SUPPLEMENT**

| **Investigational medicinal product in clinical trials** | **Centers with manufacturing license, n (%)** |
| --- | --- |
| ^68^Ga-SSTR ligands; ^68^Ga-PSMA ligands | 5 (11.6%) |
| ^18^F-PSMA ligands; FAP ligands; Amyloid; [^18^F]FET; ^15^O water | 2 (4.6%) |
| Other, including CXCR4, Choline, [^18^F]Fluorodeoxyglucose, ^68^Ga NeoB, [^11^C]Methionine, [^11^C]MRB, (-)[^18^F]NCFHEB, [^11^C]DAS8, (+)[^18^F]NCFHEB, [^11^C]FLB457, [^18^F]Fluoride, [^18^F]PI-2620, [^11^C]SCH23390, [^18^F]AB5380, [^18^F]Fallypride, [^18^F]Fluspidine, [^18^F]NAV4694 | 1 (2.3%) |

**Supplementary Table 1: Manufacturing license.** Centers per radiotracer with permission to use compounds as an investigational medicinal product in clinical trials.

**Survey**

**
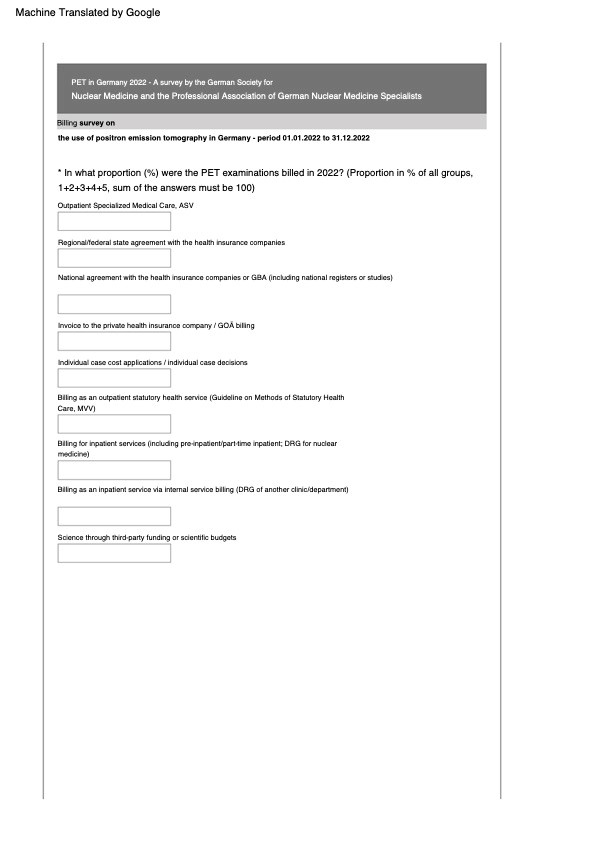
**
